# Intelligent approach to detecting online fraudulent trading with solution for imbalanced data in fintech forensics

**DOI:** 10.1038/s41598-025-01223-8

**Published:** 2025-05-23

**Authors:** Saad M. Darwish, Amr Ibrahim Salama, Adel A. Elzoghabi

**Affiliations:** 1 Department of Information Technology, Institute of Graduate Studies and Research, Alexandria University, 163 Horreya Avenue, El Shatby, P.O. Box 832, Alexandria, 21526 Egypt; 2 College of Computers and Data Science, Alexandria University, Postal code 21568, Alexandria, Egypt

**Keywords:** Fintech forensics, Detecting online fraudulent, Optimization-based sampling, Imbalanced transaction data, Electrical and electronic engineering, Computer science, Information technology, Statistics

## Abstract

Detecting online fraudulent trading in the realm of Fintech presents several challenges, primarily due to the dynamic nature of financial markets and the evolving tactics of fraudsters. Traditional machine learning algorithms trained on unbalanced datasets tend to bias towards the majority class (legitimate transactions) and may overlook minority class (fraudulent transactions) patterns. This bias can lead to poor performance in detecting fraudulent activities. The choice of sampling technique (e.g., oversampling, undersampling, SMOTE) can significantly impact model performance. However, selecting the appropriate sampling strategy requires domain knowledge and experimentation, which can be time-consuming and resource-intensive. This work utilizes Artificial Bee Colony (ABC)-based sampling to tackle class imbalance in credit card fraud detection. By generating realistic synthetic fraud samples, ABC-sampling helps the model learn fraudulent patterns more effectively without favoring non-fraudulent transactions. Inspired by the foraging behavior of bees, the process involves exploring existing fraud patterns, selecting the most relevant ones, creating synthetic fraud samples, and refining them to ensure they closely resemble real fraud cases while preserving key features that distinguish fraud from regular transactions. This method enhances fraud detection accuracy and minimizes false alarms, making the system more reliable. The suggested model employs anomaly detection algorithm to identify unusual or fraudulent trading activities in which it creates behavioral profiles for individual traders based on their historical trading activities and utilizes machine learning algorithm to cluster traders into groups based on similar behavior patterns. Then it identifies characteristic features of fraudulent traders such as sudden changes in trading volume, irregular trading hours, or trading activities inconsistent with their profile. By analyzing patterns and anomalies in traders’ behavior, these approaches can effectively identify suspicious activities indicative of fraudulent behavior. Extensive performance studies demonstrate that the proposed algorithm significantly outperforms the state-of-the-art methods by 10% in terms of accuracy.

**Supplementary Information:**

The online version contains supplementary material available at 10.1038/s41598-025-01223-8.

## Introduction

Fintech forensics investigates financial technology systems, transactions, and data to detect fraud in online banking, mobile payments, blockchain, and digital currencies. As digital transactions grow, specialized skills are essential to combat fraudulent activities and security breaches. In 2020, UK financial fraud losses were primarily due to payment card fraud (45%), authorized push payment (APP) fraud (38%), remote banking fraud (15%), and cheque fraud (1%) (https://www.formica.ai/blog/real-time-fraud-detection-in-banking-protecting-with-ai). By 2026, payment card fraud may decline to 40% with improved security, while APP fraud could rise to 42% due to real-time payments and social engineering scams. Remote banking fraud may reach 17% with expanded online banking, while cheque fraud is expected to remain minimal. These trends highlight the need for stronger security measures and fraud awareness^1,2^.

Fintech forensics employs various approaches to detect online fraud, including data analytics and machine learning, fraud detection rules, advanced identity verification, and transaction monitoring^1–4^. Machine learning models analyze large datasets to uncover hidden fraud patterns using supervised learning for classification, unsupervised learning for anomaly detection, and deep learning for complex pattern recognition. Fraud detection rules flag suspicious transactions based on predefined risk factors like amount, recipient history, and location, adapting dynamically to emerging fraud trends. Advanced identity verification enhances security through multi-factor authentication (MFA), combining biometrics with traditional methods, while AI-driven document verification detects tampering and ensures authenticity. Transaction monitoring systems analyze real-time financial activities, using AI-powered anomaly detection to identify irregularities such as rapid transactions from different locations or sudden high-value purchases, triggering alerts for further investigation.

Fintech forensics faces several challenges in detecting fraud, including the constant evolution of fraud tactics that bypass security measures, making traditional methods insufficient. Real-time transaction processing demands near-instant forensic analysis to prevent fraud effectively. Additionally, the imbalance between fraudulent and legitimate transactions can skew machine learning models, leading to high false negative rates. Concept drift further complicates detection, as fraud patterns change over time, requiring continuous model updates to maintain accuracy^1,3,4^. Handling imbalanced data in machine learning involves techniques like undersampling, oversampling, and Synthetic Minority Over-sampling Technique (SMOTE)^5–9^. Undersampling reduces the majority class size, improving efficiency but risking overfitting with excessive reduction. Oversampling balances data by replicating minority class instances, though it increases training time and memory usage. SMOTE generates synthetic samples to address imbalance without duplication but may introduce noise, particularly in high-dimensional spaces, and is sensitive to parameter selection^10,11^. Evolutionary techniques, like genetic algorithms (GAs) and evolutionary strategies (ES), offer alternative solutions for handling imbalanced data by optimizing sampling strategies and classifier ensembles. Inspired by natural selection, these methods evolve sampling approaches to balance class distribution while minimizing overfitting. They also enhance classification by generating diverse models and combining predictions through bagging or boosting^12^.

### Problem statement

Detecting fraudulent trading in Fintech remains a significant challenge due to the inherent imbalance in transaction datasets, where fraudulent activities constitute only a small fraction of overall trades. Traditional anomaly detection methods struggle with this imbalance, often biasing predictions toward legitimate transactions while failing to accurately identify fraudulent ones. Additionally, the choice of sampling techniques to address class imbalance, such as oversampling, undersampling, or synthetic data generation, requires careful tuning and domain expertise. Without a robust approach to handling class imbalance and effectively distinguishing fraudulent behavior, detection systems may suffer from high false negatives, allowing fraudulent transactions to go undetected. Furthermore, many existing approaches rely on single-stage anomaly detection, which may not effectively capture subtle fraudulent patterns hidden within highly dynamic trading environments. Single-stage approaches often rely on rigid decision boundaries or static thresholds that struggle to adapt to evolving fraud tactics. Without multi-view analysis, single-stage models fail to capture the complex, multi-dimensional nature of fraudulent activities, making them less effective in dynamic financial environments where fraudsters continuously refine their strategies.

### Motivation of the work

One of the significant challenges in detecting online fraudulent trading for fintech forensics applications is dealing with imbalanced datasets. In real-world scenarios, fraudulent transactions often represent a minority class compared to legitimate transactions. Fraudulent trading activities may evolve over time, making it challenging to collect sufficient labeled data for training robust detection models, especially for rare or emerging fraud patterns. While approaches for detecting online fraudulent trading under imbalanced datasets have shown promise, they also have several disadvantages and limitations. Some approaches for handling imbalanced datasets, such as oversampling or ensemble methods, can be computationally intensive and inefficient, particularly for large-scale online trading platforms. This can result in increased processing time and resource requirements, limiting real-time fraud detection capabilities. Furthermore, identifying relevant features that effectively capture fraudulent behavior can be challenging, especially in complex and dynamic trading environments. Limited feature space may lead to underrepresentation of certain fraud patterns. Recent advancements in optimization algorithms are being leveraged to enhance the performance of fraud detection models in handling imbalanced datasets while minimizing false positives and false negatives.

### Contribution and methodology

The aim of the work presented in this paper is to present an intelligent approach for detecting online fraudulent trading with imbalanced datasets, especially in identifying relevant features that effectively capture fraudulent behavior. Custom behavior analysis allows for the creation of tailored models that capture unique behavioral patterns indicative of fraudulent activities. By focusing on specific behaviors relevant to online trading fraud, instead of focusing solely on transactional data, these models can achieve higher detection accuracy compared to generic fraud detection approaches. The suggested model utilizes Artificial Bee Colony optimization (ABC) technique to adaptively sample from the imbalanced dataset during training, dynamically adjusting sampling weights based on the current state of the model. This can help address issues such as data scarcity in the minority class or concept drift over time.

The novelty lies in integrating optimization technique into the fraud detection process, aiming to improve the performance of machine learning model specifically tailored for imbalanced data. By introducing optimization method, the proposed approach offers a fresh perspective on mitigating the challenges posed by class imbalance that include scalability issues, computational complexity, interpretability concerns, and the need for continuous adaptation to evolving fraud patterns. The utilized optimization-based sampling algorithm prioritize samples that contribute the most to the model’s learning process, particularly those that are challenging to classify correctly due to their proximity to minority class samples or their influence on decision boundaries. Furthermore, it considers factors such as sample difficulty, proximity to minority class samples, and potential impact on decision boundaries when determining sample informativeness.

The rest of this paper is detailed as follows: Sect. 2 provides a literature survey of works related to detecting online fraudulent trading. Section 3 presents the proposed approach to detecting online fraudulent trading with extremely imbalanced data for FinTech forensics applications. Section 4 reports the evaluation of the proposed model, along with the results and the discussion. Finally, Sect. 5 contains the conclusion of the work and the directions that could be taken in future works.

## Related work

Detecting online fraudulent trading involves various methodologies, each with strengths and limitations. Rule-based systems rely on predefined fraud patterns, offering interpretability and regulatory compliance but requiring continuous manual updates to adapt to evolving tactics^13–15^. Machine learning (ML) models, whether supervised, unsupervised, or semi-supervised, can detect complex fraud patterns and process large datasets efficiently. However, they may suffer from interpretability challenges, reliance on high-quality labeled data, and overfitting risks^16–18^. Anomaly detection techniques identify deviations from normal behavior, making them effective for uncovering new fraud patterns with minimal labeled data. Yet, distinguishing between genuine anomalies and noise remains a challenge, impacting accuracy^19^. Other advanced approaches include network analysis, which examines transaction relationships using graph-based algorithms to improve detection accuracy in high-volume environments^20^. However, it demands extensive data integration and raises privacy concerns. Hybrid approaches combine multiple techniques to enhance fraud detection while reducing false positives. Despite improved accuracy, these approaches add system complexity, making maintenance and interpretability more challenging.

An effective design method for intrusion detection systems (IDS) with relation to online applications is suggested in Ref^14^. On order to combat money laundering systems, the authors created a novel set of features rooted on time-frequency analytics. These features use 2-D models of monetary processes. They used a random forest for classification and a clustering technique for hyperparameter tuning. An unsupervised, scalable method that represents manipulators’ actions in a transaction stream was introduced in Ref^21^. Rather than relying on supervised and semi-supervised learning techniques, this methodology converts real-time trades into a stream of graphs. By analyzing density signals in these graphs, fraudulent traders may be identified. From this perspective, they introduced a new metric—graph topology, time, and behavior—and exposed the characteristics of fraudulent market participants. The next step was to optimize the suggested metric greedily in order to find suspicious blocks.

The Egret Swarm Optimization technique (ESOA), a newly developed meta-heuristics technique, was used in conjunction with a machine learning approach to create a fraud detection framework in Ref^22^. Afterwards, a loss function and cost-sensitive objective function were built. The expected outcomes were then mapped into the non-linear model using the labels 0 (non-fraud) and 1 (fraud). In Ref^23^., the authors introduced a technique for detecting fraud that uses machine learning and memory compression methodology to improve detection. They presented a new nonlinear embedded machine learning base autoencoding layered approach for fixing dataset imbalances and a machine learning network for detecting fraudulent transactions.

Inadequate transaction representation, noisy labelling, and data imbalance are some of the difficulties that arise while developing machine learning-based methods for fraud detection. Also, real-world issues like verification delay, concept drift, and dynamic thresholds must be properly handled. Credit card transactions may be correctly described using a set of spatial and temporal representative features that the authors of the research reported in Ref^24^. developed. In addition, a number of supplementary self-supervised objectives were created to simulate the sequences of cardholder behavior. Intelligent sampling procedures were used to remove labels that may have been noise, which helped to reduce the amount of data imbalance.

In order to quickly build a prediction model for credit card fraud detection, the authors of Ref^25^. used Just-Add-Data (JAD), a system that automates the selection of machine learning algorithms, the tuning of their hyper-parameter values, and the estimation of performance in detecting fraudulent transactions using a highly unbalanced dataset. The user is not prompted to configure any of the parameters of the techniques during model training. Using subgraph generators and neighborhood samplers to deal with category imbalance, the authors of Ref^26^. developed a model for fraud detection based on graph neural networks. In order to better use the hidden information in transaction data and decrease the artificiality of fraudulent nodes, the model also uses a self-attentive module that distinguishes between homogeneous and heterogeneous connections.

In Ref^27^., the authors offered a credit card-not-present fraud detection and prevention (CCFDP) approach for detecting and preventing credit card not present (CNP) fraud using big data analytics. Both the fraud detection process (FDP) and the fraud prevention process (FPP) are components of the newly implemented CCFDP that work together to address suspicious conduct. First, the FDP checks the system for malicious conduct, and then the FPP helps stop it. The FDP stage employs five state-of-the-art methods: logistic regression learning (LRL), singular value decomposition (SVD), principal component analysis (PCA), t-distributed stochastic neighbor embedding (RU), and t-SNE. It is necessary for the FDP to balance the dataset in order to undertake experiments. Random undersampling is used to circumvent this problem. In addition, FDP needs to reduce the dimensionality features for better data display.

A credit card fraud forensic detection model using sequential data modelling with Long Short-Term Memory (LSTM) DNNs was introduced in Ref^28^. To make the LSTM-attention model work, we use attention mechanisms to boost the model’s performance, uniform manifold approximation to choose the most important predictive features, and transaction sequences. What makes this study stand out is its use of an LSTM-attention method to identify cases of credit card fraud and demonstrate how the model might help banks reduce the occurrence of fraudulent transactions. In Ref^29^., the presented approach is to integrate the strength of three methods: a resampling technique to deal with imbalanced data, the XGBoost technique for classification, and the differential evolution algorithm (DE) for hyperparameter selection. The fraudulent transactions are classified using the optimized XGBoost algorithm.

Using a GA to improve the hyperparameter and comparing it with grid search (GS) approaches, the authors of Ref^30^. used machine learning to identify fraudulent transactions. Classifiers such as support vector machine (SVM), decision tree (DT), logistic regression (LR), and random forest (RF) are used. They used undersampling to manage the problem of the credit card fraud dataset being highly skewed, which is an imbalanced data set. Since the sampling methodology has a significant impact on the effectiveness of fraud detection, this was their chosen method. Compared to the GS algorithm, the genetic algorithm produces better results in less time when measuring accuracy, precision, recall, and F1_score.

The approach described in Ref^31^. assists in automatically detecting fraud, identifying hidden correlations in data, and speeding up the verification process when compared to more traditional techniques of fraud detection. The Bat Optimization Algorithm (BOA) is used to identify important and distinctive features, which allows this to be accomplished. The next step is to use the synthetic minority over-sampling method (SMOTE) to balance the credit card fraud dataset, which is quite skewed. The last step in improving the performance and stability of fraud detection is to build a CNN model that uses the full center loss function to identify anomalies in credit card data. The suggested model achieves an accuracy of around 99% when tested on the Kaggle dataset.

In Ref^32^., inspired by generative adversarial networks (GANs), the authors introduced BalanceGAN, a GAN-based approach for detecting online banking fraud on highly unbalanced data. A model for fraud detection is trained using the generator’s data, and then it is fine-tuned using transfer learning using real-world datasets to fix data imbalances. When it comes to addressing unbalanced data, BalanceGAN is superior to traditional approaches since it prevents the model from being overfit.

To tackle the problem of identifying fraudulent online trading, the authors in Ref^33^. presented a new method that integrates autoencoder (AE) and fully connected deep network (FCDN) models. There are three steps to this process: first, training and AE on fraudulent transactions; second, using another AE to reduce dimensionality; and last, training FCDN on the encoded representations. They added another FCDN that was trained on the preprocessed data using the synthetic minority oversampling method (SMOTE) to boost the model’s performance even more. In Ref^34^., the authors utilized two XGBoost and Fully Connected Neural Network–based fraud detection algorithms to tackle the challenge of difficult fraud detection in network transactions.

For the purpose of detecting financial fraud, a comparison analysis of four Quantum Machine Learning (QML) models was carried out in Ref^35^. With F1 scores of 0.98 for both the fraud and non-fraud classes, the authors demonstrated that the Quantum Support Vector Classifier model attained the maximum performance. The promise of QML classification for financial applications is being propelled by other models that show promising results, such as the Variational Quantum Classifier, Estimator Quantum Neural Network (QNN), and Sampler QNN. However, there are obstacles, such as the need for quantum algorithms that are more efficient and for datasets that are both bigger and more complicated.

Three models were defined in Ref^36^. : a risk model to forecast the likelihood of fraud while taking countermeasures into account, an economic optimization of machine learning results, and a machine learning-based fraud detection model. Actual data was used to test the models. In comparison to a static if-then rule benchmark, their machine learning model alone reduces expected and unexpected losses in the three pooled payment channels by 15%. The expected losses are reduced by 52% after further optimizing the machine-learning model. The results remain valid even when the false-positive rate drops to 0.4%. In light of this, the three models’ risk frameworks are practical from both a business and risk standpoint.

Using both publicly accessible and real-life transaction records, the authors of Ref^37^. applied a total of thirteen statistical and machine learning models to identify fraudulent charges on payment cards. Both the original features and the aggregated features are investigated and compared based on the results. To determine whether the combined genetic algorithm features can provide more discriminative power in fraud detection than the original features, a statistical hypothesis test is performed. The results showed that aggregated features work well for detecting real-world payment card fraud. For an overview of banking fraud detection techniques such as supervised, unsupervised, optimization, and hybrid approaches, the reader can refer to^17,38–51^. Table 1 provides a clear comparison of the different approaches, their strengths, and potential limitations.

**Table 1:** Comparison of fraud detection techniques: strengths and limitations.

Ref.	Method	Pros	Cons
^14^	Time-frequency analytics + Random Forest	Captures temporal patterns, effective classification	Requires careful hyperparameter tuning
^21^	Unsupervised graph-based fraud detection	Identifies manipulators without labeled data	Computationally expensive
^22^	ESOA + ML-based fraud detection	Optimizes detection through meta-heuristics	Complexity in model tuning
^23^	Memory compression + Autoencoding ML	Reduces dataset imbalance	High memory and processing overhead
^24^	Spatial-temporal feature representation	Improves fraud detection accuracy	Sensitive to noisy labels
^25^	Just-Add-Data (JAD) automated ML	No manual tuning required	Model interpretability is limited
^26^	Graph Neural Networks (GNN)	Leverages transaction relationships	Sensitive to graph construction quality
^27^	Big data analytics + FDP & FPP	Combines detection and prevention	High data preprocessing requirement
^28^	LSTM + Attention for sequential fraud detection	Captures long-term dependencies in transactions	Requires large training data
^29^	XGBoost + Resampling + DE	Effective for imbalanced data	Computationally expensive
^30^	Genetic Algorithm (GA) + ML classifiers	Improves classification accuracy	Model training time increases
^31^	Bat Optimization Algorithm (BOA) + CNN	High accuracy with optimized features	Computationally expensive
^32^	BalanceGAN for fraud detection	Handles data imbalance effectively	Requires fine-tuning of GAN models
^33^	AE + FCDN + SMOTE	Enhances detection accuracy	Computationally intensive
^34^	XGBoost + Fully Connected Neural Networks	Efficient and accurate	High complexity
^35^	Quantum Machine Learning (QML)	Promising results for fraud detection	Limited by quantum hardware constraints
^36^	Risk modeling + ML-based optimization	Reduces financial losses	Requires business-specific customization
^37^	Statistical + ML-based fraud detection	Effective feature aggregation	Feature selection is critical

While existing fraud detection methods employ a range of machine learning techniques—including supervised, unsupervised, and hybrid models—many face critical limitations such as reliance on imbalanced datasets, sensitivity to noisy labels, lack of adaptability to evolving fraud tactics, and computational inefficiency. Approaches like random forests and XGBoost show strong classification performance but struggle with generalization in dynamic financial environments. Deep learning techniques such as LSTMs and GANs improve representation learning but often require extensive training data and suffer from interpretability issues. Optimization-based methods, including ESOA and genetic algorithms, enhance feature selection but may lead to suboptimal local minima in complex fraud detection scenarios. Quantum machine learning models exhibit promising accuracy but remain constrained by hardware limitations and practical scalability.

### Limitations of existing literature

Extending the related work on detecting online fraudulent trading with imbalanced data involves exploring and incorporating new methodologies, or datasets to enhance the effectiveness of fraud detection systems. Some avenues for extension in our case include exploring a new set of features derived from transactional data to capture subtle patterns indicative of fraudulent behavior. Custom behavior analysis enables the extraction of features that capture the contextual nuances of online trading. This contextual understanding can help identify the most informative features and enhance the accuracy of fraud detection models. Furthermore, investigate swarm optimization- based sampling technique to address class imbalance. ABC technique is applied to optimize the parameters of undersampling to generate synthetic or balanced datasets for model training. By optimizing certain criteria, such as diversity or coverage, ABC technique can select samples that better represent the entire dataset with fewer samples compared to traditional random or systematic sampling methods. ABC methods are robust to noisy objective functions or fitness landscapes. This robustness is due to their stochastic nature and ability to maintain diversity within the population of candidate solutions. As a result, ABC algorithms can effectively handle optimization problems in noisy or uncertain environments, where traditional methods may struggle. ABC algorithms typically have fewer control parameters compared to other optimization techniques, reducing the need for extensive parameter tuning. This simplicity makes them more accessible and easier to use for practitioners, particularly in applications where domain expertise may be limited.

## The proposed model

Detecting fraud requires understanding an account’s normal behavior through specific indicators and identifying significant deviations. This involves three key steps: identifying the most relevant transaction features for distinguishing legitimate from fraudulent activity, characterizing account behavior using these features, and determining criteria for issuing alarms when fraud is likely. Profiles are developed through a training phase, where normal behavior is established based on non-fraudulent activity, and a usage phase, where deviations from this baseline are assessed. The goal is to automate the creation of user-profiling systems for reliable fraud detection.

**Fig. 1:**
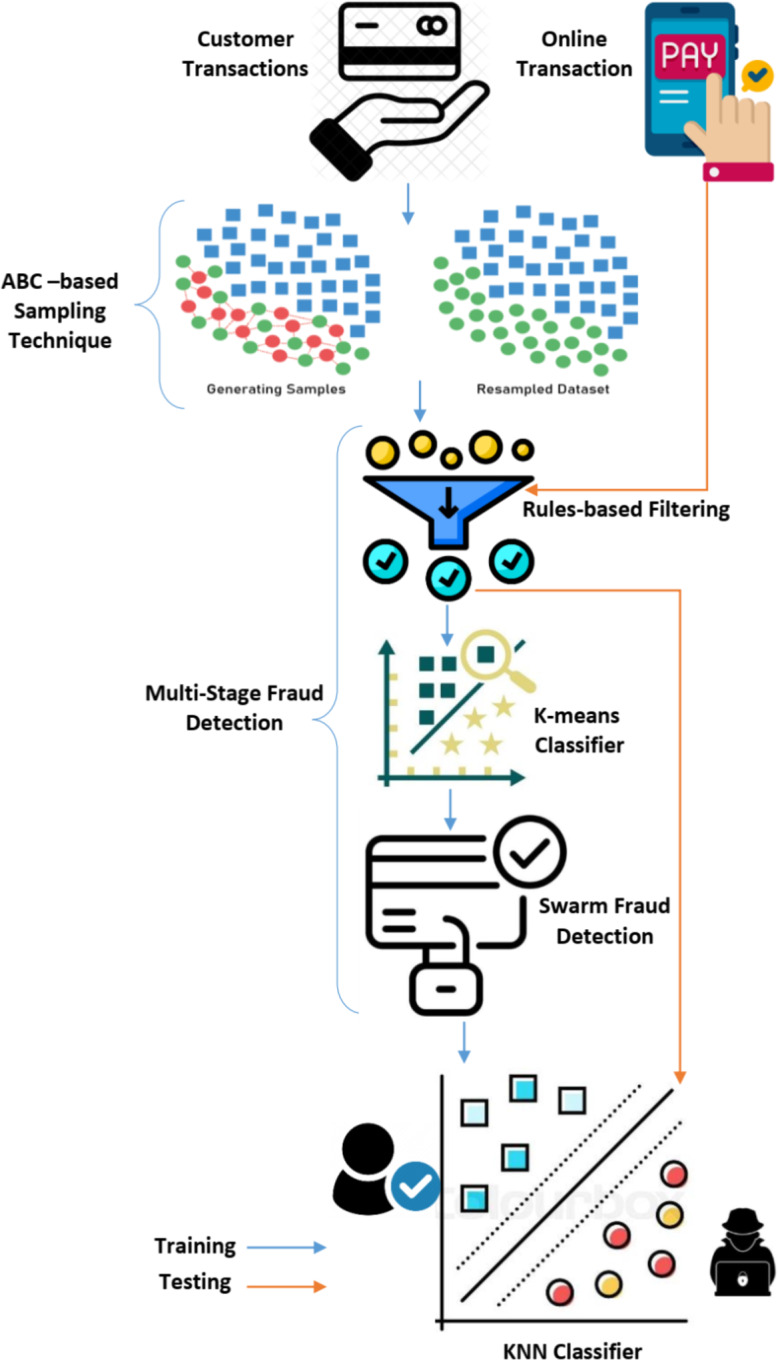
The proposed multi-stage fraud detection model.

The proposed model employs multi-stage classification approach that combines rule-based filtering, K-means clustering, an Artificial Bee Colony (ABC)-based classifier and K-Nearest Neighbors Classifier in a unified framework, which seeks to optimize fraud detection while minimizing false alarms. The suggested model poses two distinct phases. In the first phase, starting with raw transaction attributes in the initial rule-based filter, it advances to the level-one K-means classifier, which uses derived critical values from transactions. In level two, the ABC-based classifier employs a structured dataset comprising seven Boolean values, which include five critical values, the level-one classification result, and historical classifications. This layered data fusion culminates in an integrated decision-making process, enhancing the model’s accuracy in detecting fraud. The second phase is the detection phase, where a profile is created for the customer based on the new incoming transaction. This profile is then compared to the stored spending profile of the cardholder using the KNN classifier. Figure 1 shows the main system components and how they are linked to each other.

The integration of K-means and ABC optimization is achieved through semantic decision-level fusion, enabling combined results from both classifiers to yield higher-quality estimates than either could individually. This fusion addresses the challenge of linking variables from each clustering level to meaningful human interpretation. In this model, each classifier receives input derived from transaction attributes, and factors critical to ABC performance—such as the local search process and a parameter-based fitness function—are optimized in the first fusion level. The following subsections detail each component involved in the training and testing phases.

### Phase 1: training phase

The training phase generates an expenditure profile $\:P$ for each cardholder based on past transactions. This profile is created by applying K-means and ABC-based classification on filtered transaction data. Let $\:T$ is the transaction data for a particular cardholder, $\:X=\{{x}_{1},{x}_{2},\dots\:,{x}_{N}\}$ is the raw attributes of a transaction, $\:{C}_{i}$ is the classification result at classifier level$\:\:i$, and $\:P$ represents the stored expenditure profile of the cardholder (created during the training phase).

### **Step 1: ABC-Sampling for balancing imbalanced datasets**

In the context of online trading, fraudulent transactions represent a small portion of the total transaction volume, leading to a highly imbalanced dataset. The challenge is to build a classifier capable of detecting fraud accurately while managing this class imbalance to minimize both false negatives (missed fraud) and false positives (legitimate transactions flagged as fraud). Assume a dataset $\:D$ containing $\:N$ transactions, each represented by $\:{X}_{i}\in\:{\mathbb{R}}^{d}$ that is the feature vector for the $\:i$-th transaction, consisting of$\:\:d$ attributes (e.g., transaction amount, frequency, user activity metrics) and $\:{y}_{i}\in\:\left\{\text{0,1}\right\}$ that is the label for the transaction, where $\:{y}_{i}=1\:$indicates a fraudulent transaction and $\:{y}_{i}=0$ indicates a legitimate transaction. This dataset can be split into two subsets:

1 $$\:D={D}_{0}\cup\:{D}_{1}\:$$,

where $\:{D}_{0}$consists of legitimate transactions and$\:{D}_{1}$​ consists of fraudulent ones, with $\:\left|{D}_{1}\right|\ll\:\:\left|{D}_{0}\right|$​, reflecting the high class imbalance. The primary objective is to design a classifier $\:f:{\mathbb{R}}^{d}\to\:\left\{\text{0,1}\right\}$ that maximizes fraud detection accuracy while balancing precision and recall.

To address class imbalance, the model applies ABC-driven swarm optimization to generate a balanced dataset. This technique selects a representative subset $\:\stackrel{\sim}{D}\subset\:D$ with enhanced diversity and coverage, achieved by optimizing either undersampling or oversampling:

2 $$\:\stackrel{\sim}{D}=arg\:\underset{\acute{D}}{\text{max}}\left(Diversity\right(\acute{D})+Coverage\:(\acute{D}\left)\right)$$,

This results in a more balanced training set, ensuring that the model encounters both legitimate and fraudulent behaviors, improving robustness and generalization. Diversity measures how varied the selected subset $\:\acute{D}$ is in terms of feature distribution and class representation. A diverse subset should capture a broad range of feature values, transaction types, and patterns present in the original dataset $\:D$. Maximizing diversity ensures that the selected subset $\:\stackrel{\sim}{D}$ includes examples that represent different transaction behaviors, both legitimate and fraudulent. This is particularly important in detecting fraud, as fraudulent behavior can vary widely. Coverage assesses how well the subset $\:\acute{D}$ includes critical examples that represent the most relevant patterns and behaviors in the dataset. In fraud detection, coverage is vital to ensure that typical fraudulent transaction profiles are included. Maximizing coverage ensures that the subset $\:\stackrel{\sim}{D}$ captures essential characteristics or patterns that the classifier will encounter in real-world data, making the model more robust and generalizable.

ABC (Artificial Bee Colony) sampling is a robust technique for handling imbalanced datasets by generating synthetic samples to augment minority classes—in this case, fraudulent transactions^52,53^. Here’s how ABC optimization steps can be linked to fraud detection for balancing a dataset in a banking fraud detection system.

- *Initialization*: Let $\:{X}_{i}=\left\{{x}_{i1},{x}_{i2},\dots\:\dots\:,{x}_{id}\right\}$ represent a transaction in the minority class (fraudulent). Initialize$\:\:N$ food sources from fraudulent transactions as$\:\:X=\{{X}_{1},{X}_{2},\dots\:.,{X}_{N}\}$ where $\:d$ is the number of features in each transaction.
- *Fitness Function*$\:\:F\left(X\right)$: The fitness function evaluates how well a synthetic transaction represents a real fraudulent transaction. A common choice is to use a distance metric, such as Euclidean distance, based on the closeness of transaction features to the original minority class:

3 $$\:F\left({X}_{i}\right)=\frac{1}{1+dist\left({X}_{i},{Centroid}_{fraud}\right)}$$

where $\:{Centroid}_{fraud}$ is the centroid of the fraudulent transactions in the feature space. Higher fitness values indicate more realistic fraudulent transactions.

- *Employed Bee Phase*: Each employed bee modifies the current food source (synthetic transaction) $\:{X}_{i}$ by exploring neighboring values, creating a new transaction$\:{\:V}_{ij}$ as:

4 $$\:{V}_{ij}={\text{X}}_{ij}+{\varphi\:}_{ij}\left({\text{X}}_{ij}-{\text{X}}_{kj}\right)$$

$\:{\text{X}}_{ij}$​ is the $\:j$-th feature of the $\:i$-th transaction, $\:{\varphi\:}_{ij}$ is a random number between − 1 and 1, and $\:{\text{X}}_{kj}$ is a randomly selected transaction from the fraudulent class,$\:\:k\ne\:1$. If the fitness $\:F\left({V}_{ij}\right)>F\left({X}_{i}\right)$ replaces $\:{X}_{i}$as a better synthetic fraudulent transaction.

#### Onlooker bee phase

Onlooker bees choose food sources based on their fitness probability $\:{P}_{i}$​

5 $$\:{P}_{i}=\frac{F\left({X}_{i}\right)}{{\sum\:}_{j=1}^{N}F\left({X}_{i}\right)}$$

Higher fitness food sources have a higher probability of being selected for further synthetic transaction generation. This focuses the sampling on more representative fraudulent examples.

- *Scout Bee Phase*: If a food source $\:{X}_{i}$​ does not yield improved fitness over a certain number of trials (a parameter known as “limit”), it is abandoned, and a new food source $\:{X}_{i}$​ is randomly initialized:

6 $$\:{X}_{i}=random\:initialization\:in\:feature\:space$$

- Stopping Criteria: ABC sampling continues until the minority class size reaches a targeted ratio with respect to the majority class, achieving dataset balance. If $\:{N}_{fraud}$​ and $\:{N}_{genuine}$​ are the sizes of fraudulent and genuine classes, respectively, the algorithm stops when:

7 $$\:\frac{{N}_{fraud}}{{N}_{genuine}}\ge\:Target\:Ratio$$

This pseudocode listed in Appendix- Algorithm 1 provides a structured representation of the ABC Sampling Technique, ensuring diversity and coverage of the synthetic samples to address class imbalance effectively.

### **Step 2: Rule-based filtering**

The rule-based engine plays a crucial role in the transaction classification process by acting as an initial filter that identifies potentially fraudulent activities with a certain probability. It evaluates incoming transactions by assessing the extent to which their behavior deviates from the normal profile of the cardholder. This filtering process incorporates both generic and customer-specific rules, such as average daily or monthly spending patterns and discrepancies between shipping and billing addresses^54,55^. Initially, simple rule-based filtering is applied to reduce data volume by removing transactions that meet predefined criteria. Let $\:{X}_{i}^{filtered}\:$denote the transactions passing the filter:

3 $$\:{X}_{i}^{filtered}=\left\{{X}_{i}\in\:D|\:meet\:criteria\right\}$$

The database consists of records of customers’ credit card transactions, with each transaction defined by a specific set of attributes, as detailed in Table 2. These attributes are processed by a rules engine, which applies a predefined set of rules to analyze the data. Through this analysis, the rules engine generates five key filtered parameters, outlined in Table 3, which are used to calculate critical values for each transaction. These critical values act as indicators for an initial classification, categorizing the transaction as either potentially fraudulent or normal. These rules leverage critical values$\:\:\delta\:\:$derived from key transaction parameters to provide a preliminary classification as listed in Appendix- Algorithm 2.

### **Step 3: K-mean classifier (level 1 classification)**

In this system, each transaction is associated with a data structure consisting of five Boolean values $\:{{\updelta\:}}_{{T}_{i,j}}$. These values indicate whether each transaction operation is classified as fraudulent (suspicious) or legitimate (unsuspicious) based on critical thresholds established by the rule-based filtering engine. This preliminary classification serves as input for the first layer of an advanced classification system that utilizes the *k*-means clustering algorithm. The incorporation of this additional level of filtering is critical, as it addresses the inherent limitations of rule-based filtering systems, such as their rigidity and potential inability to adapt to complex, evolving fraud patterns.

The *k*-means algorithm, begins by selecting *k* initial centroids, where *k* represents the number of desired clusters. In the context of CCFD, the algorithm is designed to identify two clusters: one representing potentially fraudulent transactions and the other representing legitimate transactions. Each transaction, encoded as a data point from the Boolean values, is assigned to the cluster of the closest centroid based on a proximity measure. For this purpose, the Euclidean distance is employed, as it effectively quantifies the similarity between a data point and the cluster centroids in a multi-dimensional space^56,57^.Once all transactions are allocated to their respective nearest centroids, clusters are formed. The centroids of these clusters are then recalculated as the mean of all points within each cluster. This process iteratively assigns transactions to clusters and refines the centroid positions, continuing until cluster memberships stabilize and no further changes occur. The output of this level is $\:{}_{Model,{T}_{i,j},\:l1\:\:}$that represent the trained clustering model at level 1.

The significance of this *k*-means-based classification lies in its ability to dynamically group transactions with similar characteristics, overcoming the static and predefined nature of rule-based systems^57^. While the initial rule-based filtering serves to establish a baseline categorization, it is susceptible to misclassification due to its reliance on rigid thresholds that may not encompass the precise of real-world fraud behavior. By introducing a clustering mechanism, this second layer provides a more adaptive and data-driven approach, ensuring that transactions are grouped based on their underlying patterns rather than pre-established rules alone. This enhances the overall accuracy and robustness of the fraud detection system, particularly in identifying emerging or sophisticated fraudulent activities that might bypass the initial rule-based filters.

**Table 2:** Key attributes for credit card transaction analysis.

No.	Attribute	Description	Purpose
1	Card Id	A unique identifier assigned to each credit card	Used to distinguish between transactions for different cards, enabling tracking and analysis of card-specific activity
2	Location of transaction $\:{T}_{loc}$	The geographic location where the transaction occurred	Helps identify anomalies, such as transactions made in unusual or distant locations compared to the cardholder’s typical activity zones
3	Current balance$\:\:{CC}_{cb}$	The remaining balance available on the card at the time of the transaction.	Monitors spending habits and identifies rapid depletion of the balance, which can be a sign of fraudulent activity
4	Average bank balance $\:{B}_{ab}$	The average balance maintained by the cardholder in their associated bank account over a certain period	Provides insight into the cardholder’s financial stability and helps detect significant deviations in spending behavior
5	Amount of transaction $\:{T}_{amt}$	The monetary value of a single transaction.	Used to evaluate the size of transactions, especially large amounts that may deviate from normal spending patterns
6	Credit card age $\:{CC}_{age}$	The duration of time since the credit card was issued	Helps assess the cardholder’s transaction history and provides context for typical spending patterns, as newer cards may exhibit different behaviors compared to older ones
7	Authentication type $\:{T}_{au}$	The method used to authenticate the transaction (e.g., PIN, OTP, biometric).	Ensures transaction security and helps identify potential breaches in authentication protocols
8	Location of card used $\:{CC}_{loc}$	The geographic location where the credit card is physically used	Cross-referenced with $\:{T}_{loc}$ to detect inconsistencies, such as card-not-present fraud or mismatches between the transaction and cardholder’s location
9	Number of overdraft $\:{N}_{cc\_od}$	The number of instances the cardholder has exceeded their credit limit	Indicates potential financial strain or attempts to exploit the card’s credit limit, which could be associated with fraudulent behavior
10	Overdraft transaction $\:{T}_{od}$	Specific transactions made during overdraft periods	Analyzes the nature of transactions during overdrafts, helping to distinguish between legitimate and suspicious activities
11	Card used$\:{\:T}_{cu}$	Total number of card use during a period	Regular, consistent usage patterns can indicate normal activity, while sudden increases or unusually high usage within a short timeframe might raise red flags for potential fraudulent activity
12	Card used transaction $\:{N}_{T\_cu}$	The total number of transactions conducted using the card	Helps in understanding transaction frequency and identifying sudden spikes in activity that may signal fraud

**Table 3:** Five key filtered parameters for credit card transaction analysis.

No.	Attribute	Equation	Definition	Significance
1	$\:{CC}_{Frquency}$	$\:\frac{{\:T}_{cu}}{{CC}_{age}}$	This parameter tracks the frequency of credit card usage over a specific time frame (e.g., daily, weekly, or monthly)	Unusual spikes in usage frequency can signal suspicious activity. For example, if a card is suddenly used multiple times within a short period, it could indicate fraudulent attempts, such as testing the card’s validity or conducting rapid unauthorized transactions
2	$\:{CC}_{Usage\_Location}$	$\:\frac{{CC}_{loc}}{{T}_{loc}}$	This parameter captures the geographic locations where the credit card is being used.	Transactions occurring in locations far from the cardholder’s typical activity zones, or in high-risk areas known for fraud, may indicate fraudulent use. Geolocation data is cross-referenced with the cardholder’s usual locations and flagged if anomalies are detected
3	$\:{CC}_{Overdraft}$	$\:\frac{{N}_{cc\_od}}{{\:T}_{cu}}$	This parameter measures how often and how quickly the credit limit is exceeded by the cardholder	A high overdraft rate or rapid successive overdrafts can indicate either reckless financial behavior or fraudulent exploitation of the card. Monitoring overdraft rates helps detect unusual spending behavior
4	$\:{CC}_{Balance}$	$\:\frac{{CC}_{cb}}{{B}_{ab}}$	This refers to the current available balance on the credit card, reflecting the funds left after all transactions and payments	Fraudsters often aim to deplete the card’s balance quickly. A rapid reduction in the book balance, especially without corresponding repayments, is a strong fraud indicator.
5	$\:{CC}_{Daily\_Spending}$	$\:\left(100000-{B}_{ab}\right)\times\:\left(\frac{{CC}_{age}}{30}\right)$	This parameter calculates the average amount spent daily using the credit card over a defined period.	Significant deviations from the average daily spending can highlight potential fraud. For example, an unusually high daily spending pattern may indicate unauthorized large purchases

### **Step 4: Swarm fraud detection (level 2 classification)**

The performance of the *K*-Means clustering algorithm is highly sensitive to the initial selection of centroids, which often leads to suboptimal clustering solutions. This limitation becomes particularly critical in applications such as fraud detection, where accurate classification and identification of anomalies are paramount. To address this issue, ABC algorithm has been adapted to enhance the clustering process and improve the overall detection accuracy. The ABC algorithm plays a pivotal role in improving the *K*-Means clustering process by optimizing the initial centroid selection and refining cluster assignments iteratively. This optimization is achieved through the collective behavior of three types of artificial bees—employed, onlooker, and scout—working collaboratively to explore the solution space and identify centroids that lead to more compact and well-separated clusters. By employing the fitness function, the ABC algorithm ensures that clusters not only minimize intra-cluster distances but also align with the known classification labels (real fraud or legitimate transactions).

In the adapted approach, each transaction in the dataset is represented as a data structure containing seven Boolean features. These include five critical attributes that encapsulate key transactional behaviors $\:{{\updelta\:}}_{{T}_{i,j}}=\left\{{{\updelta\:}}_{1,{T}_{i,j}},\:{{\updelta\:}}_{2,{T}_{i,j}},\:{{\updelta\:}}_{3,{T}_{i,j}},\:{{\updelta\:}}_{4,{T}_{i,j}},\:{{\updelta\:}}_{5,{T}_{i,j}}\right\}$, along with two additional Boolean values: one representing the classification type computed during the first level of classification $\:{}_{Model,{T}_{i,j},\:l1\:\:}$(Level 1 classification) and the other indicating the actual classification label derived from historical data in the training set $\:{}_{Model,real\:\:}$. The integration of Level 1 classification results and historical labels into the data structure allows the ABC algorithm to explicitly factor classification accuracy into the clustering process. This alignment between cluster formation and fraud detection objectives enables the algorithm to better separate fraudulent transactions from legitimate ones, enhancing the reliability and precision of the detection system. Here’s a high-level algorithm (see Appendix - Algorithm 3) to achieve this.

### **Step 5: K-nearest neighbors classifier**

The final-stage KNN classifier is intricately linked to the outputs of the preceding Rule-Based Filtering, K-means Clustering (Level 1 classification), and Swarm Fraud Detection (Level 2 classification), each of which refines the input for the final decision. Rule-Based Filtering acts as the initial gatekeeper, leveraging predefined rules to quickly eliminate obvious legitimate transactions and flag suspicious ones, reducing the dataset size and focusing the pipeline on potentially fraudulent cases. K-means Clustering then groups transactions into clusters based on their similarity, assigning preliminary labels to transactions based on patterns in the data, such as clusters of known fraudulent behaviors. This clustering narrows the scope for Swarm Fraud Detection, which operates at Level 2, employing swarm intelligence to optimize feature selection and detect complex fraud patterns that clustering alone might miss. The refined and enriched outputs from these stages, including filtered data and optimized feature spaces, are passed to the KNN classifier. KNN then performs precise instance-based classification, employing the contextual information derived from earlier stages to provide the final, high-confidence decision on whether a transaction is fraudulent or legitimate. This multi-stage approach ensures a robust and efficient fraud detection system where each level complements the final classifier. The synergy between KNN and the preceding levels enhances the overall robustness and precision of the fraud detection system, ensuring reliable real-time decisions^58^.

To conclude, the fraud detection framework employs a multi-stage hybrid anomaly detection approach that integrates multiple algorithms to identify fraudulent trading activities. The key anomaly detection components include:

- Rule-Based Filtering: This acts as an initial anomaly detector by identifying transactions that deviate from predefined normal behavioral profiles.
- K-Means Clustering (Level 1 Classification): A clustering-based anomaly detection technique that groups transactions into two clusters—fraudulent and legitimate—based on transaction similarity using Euclidean distance.
- Swarm-Based Fraud Detection (Level 2 Classification - ABC Algorithm): The ABC algorithm enhances K-Means clustering by optimizing the initial centroid selection and refining cluster assignments iteratively.
- KNN Classifier: The final classification step refines fraud detection by evaluating transaction similarities in a high-dimensional space, leveraging outputs from previous stages.

This hybrid approach combines unsupervised (K-Means clustering, ABC optimization) and supervised (KNN classification) learning, ensuring robust fraud detection.

### Phase 2: testing phase

For testing, the proposed model processes an online transaction by sequentially applying multiple classification stages to ensure accurate and efficient fraud detection. Initially, the model extracts transaction attributes, representing key features of the transaction. Next, it evaluates these attributes against a predefined set of fraud detection rules. This step acts as an initial filter, identifying transactions that may warrant further inspection. Subsequently, the model employs the KNN algorithm to classify the transaction by comparing its attributes to the solutions generated by the ABC algorithm during the training phase, which optimized the clustering and classification processes. The transaction is assigned a classification based on its proximity to these optimized solutions, employing the distance metric to determine closeness. Notably, while the training phase of the model (offline) requires significant computational time to process data, optimize parameters, and establish classification boundaries, the testing phase (online) is highly efficient. By consolidating the results of the rule-based and ABC-optimized KNN classifications, the model ensures rapid and reliable decision-making with minimal delay during real-time transaction processing.

### Evaluation parameters

Evaluating the performance of fraud detection models requires a set of well-defined metrics to measure their accuracy, reliability, and efficiency. These evaluation parameters help assess how effectively a model distinguishes between fraudulent and legitimate transactions. Key metrics include Accuracy (%), Precision (%), Recall (%), F1-Score (%), AUC-ROC, Average Latency (ms), and Throughput (transactions/second). Each of these parameters plays a crucial role in determining the model’s effectiveness in real-world fraud detection scenarios. Accuracy (%) calculates the proportion of correctly classified transactions out of the total samples, reflecting overall model correctness. Precision (%) indicates the proportion of correctly identified fraud cases among all predicted fraud cases, emphasizing the reliability of positive predictions. Recall (%), or sensitivity, measures the proportion of actual fraud cases correctly detected by the model, demonstrating its ability to capture fraudulent activities. F1-Score (%) balances precision and recall by computing their harmonic mean, offering a single performance measure. AUC-ROC (Area under the Receiver Operating Characteristic Curve) evaluates the model’s ability to distinguish between fraudulent and legitimate transactions across different threshold values, with higher values indicating better discrimination capability. Average Latency (ms) measures the time taken to process a transaction from input to output, reflecting system responsiveness, while Throughput (transactions/second) represents the number of transactions the system can handle per second, highlighting its efficiency. Lower latency and higher throughput are essential for real-time fraud detection to ensure timely and accurate decision-making^17,19,27^.

Furthermore, feature importance analysis is crucial in fraud detection as it identifies which transaction attributes contribute most to accurate classification. Importance Score quantifies the contribution of each feature to the model’s decision-making process, allowing prioritization of the most influential variables. Features with high importance scores significantly enhance classification accuracy by providing strong distinguishing patterns between fraudulent and legitimate transactions. Additionally, Correlation with Classification Outcomes examines how individual features relate to fraud labels, ensuring that highly correlated features improve model performance without introducing noise or redundancy. Strongly correlated features enhance the model’s ability to detect anomalies, while weak or irrelevant features can negatively impact classification effectiveness. A well-optimized feature selection process improves fraud detection accuracy and ensures robust model performance^30,31^.

## Experimental results

To validate the proposed model for detecting online fraudulent trading in the realm of Fintech, many experiments were conducted. These experiments aim to evaluate the model’s robustness, effectiveness, and generalization capability while addressing the specific challenges of class imbalance, anomaly detection, and fraud classification. The experiments were conducted on a system equipped with an Intel^®^ Core™ i5 processor with 8 GB RAM, implemented in MATLAB R2015a. The evaluation utilized a real e-commerce dataset comprising transactions from 99 customers, with 927 transactions for training and 100 transactions for testing. The dataset included 13 attributes: 12 features (as detailed in Table 1) and a classification label. To address the inherent class imbalance, an ABC-sampling technique was employed, which leverages the collective intelligence of artificial bees to generate synthetic samples. By iteratively refining feature importance and constructing solutions guided by pheromone trails, ABC-sampling ensures a balanced dataset while preserving critical discriminative information for classification. A synthetic dataset with 100,000 transactions, generated using C# programming and featuring the same attributes, was utilized to assess the model’s accuracy and scalability with a larger database.

### *Experiment 1: Data preprocessing and class imbalance evaluation*

This experiment aims to evaluate the performance of the utilized ABC-sampling approach in addressing class imbalance compared to traditional sampling methods like SMOTE, oversampling, and undersampling. The evaluation will focus on metrics such as accuracy, precision, recall, and F-measure to determine the effectiveness of each method in detecting fraudulent transactions. The process involves data preprocessing, applying sampling techniques, model training, and testing. The performance outcomes of the different sampling techniques for fraud detection are detailed in Table 4. The results indicate that the model without any sampling (No Sampling) performs the worst, achieving only 74.2% accuracy, 50.7% recall, and an F-measure of 58.2%. The low recall suggests that the model struggles to correctly identify minority class instances, which is expected due to class imbalance. Oversampling achieves moderate results (Accuracy: 82.5%, F-Measure: 67.7%) by replicating minority samples, which improves recall but risks overfitting and fails to capture diverse fraudulent patterns. Undersampling, by reducing the majority class, enhances precision (74.1%) but significantly lowers recall (53.4%) due to the loss of valuable legitimate transaction data. SMOTE, which generates synthetic minority samples through interpolation, shows marked improvement across all metrics (Accuracy: 88.1%, F-Measure: 79.5%), striking a better balance between precision and recall. However, the reliance on synthetic data may introduce noise.

**Table 4:** Performance comparison of sampling techniques for fraud detection.

Sampling Technique	Accuracy	Precision	Recall	F-Measure
No Sampling	74.2%	68.5%	50.7%	58.2%
Oversampling	82.5%	70.4%	65.2%	67.7%
Undersampling	76.3%	74.1%	53.4%	62.1%
SMOTE	88.1%	81.7%	77.4%	79.5%
ABC-Sampling	**95.7%**	**89.2%**	**85.6%**	**87.3%**

ABC-Sampling outperforms other techniques due to its advanced optimization-driven approach to data balancing. Unlike traditional oversampling or undersampling, ABC-Sampling is inspired by the foraging behavior of honeybee swarms, utilizing intelligent exploration and exploitation strategies to optimize sample selection. This technique strategically generates synthetic data points while preserving the structural integrity of the minority class distribution, mitigating issues such as overfitting or loss of information. Compared to SMOTE, which relies on linear interpolations between minority class samples, ABC-Sampling dynamically searches for optimal synthetic instances, ensuring greater diversity in the generated data. The result is a more balanced dataset with improved model generalization, as reflected in the significantly higher accuracy (95.7%), precision (89.2%), recall (85.6%), and F-measure (87.3%).

Another key technical advantage of ABC-Sampling lies in its adaptive selection mechanism, which differentiates between high-quality and redundant synthetic samples. While traditional resampling methods like oversampling or SMOTE risk introducing noisy or unrealistic data points, ABC-Sampling applies an iterative optimization process where artificial bees explore candidate solutions (synthetic samples), evaluate their effectiveness, and adjust sampling strategies accordingly. This self-adaptive nature enhances the model’s ability to learn meaningful decision boundaries, reducing bias toward the majority class and improving recall without compromising precision. Furthermore, ABC-Sampling’s capability to dynamically refine sample distributions makes it particularly effective in complex, high-dimensional datasets, offering superior performance in fraud detection where data imbalance is a major challenge.

### *Experiment 2: Feature importance and selection analysis*

The objective of this experiment is to analyze the feature importance scores used by the ABC-sampling algorithm and evaluate how these scores influence the generation of synthetic samples for fraud detection. By assessing the correlation between selected features and classification outcomes, we aim to ensure that the synthetic samples generated align with the critical features required for accurately detecting fraudulent activities. The experiment will also assess the impact of each feature’s importance on the quality of the synthetic samples produced, helping to fine-tune the sampling process and improve the overall fraud detection model’s performance.

The feature importance analysis in the Table 5 reveals the relative contribution of different attributes in predicting fraudulent transactions. “Amount of Transaction” and “Location of Transaction” have the highest importance scores (0.35 and 0.32, respectively), indicating their strong influence on detecting fraudulent activities. These features exhibit high correlation with classification outcomes (+ 0.88 and + 0.85), making them critical for generating accurate synthetic samples. Similarly, “Current Balance” (0.28) and “Card Used” (0.25) also show high importance and strong correlation, suggesting that they are essential in detecting fraud. On the other hand, features like “Number of Overdraft” (0.10) and “Location of Card Used” (0.12) have lower importance and weaker correlations, indicating their minimal impact on fraud detection and synthetic sample quality. Features with moderate importance, such as “Average Bank Balance” (0.22), “Credit Card Age” (0.15), and “Authentication Type” (0.18), play a supporting role in refining the synthetic samples but are less influential than the high-impact features. Overall, the table demonstrates that focusing on high-importance features will lead to better synthetic sample generation and more accurate fraud detection outcomes.

**Table 5:** Feature importance analysis and its impact on synthetic sample quality for fraud detection.

Feature	Importance score	Correlation with classification outcomes	Impact on synthetic sample quality
Location of Transaction	**0.32**	**+ 0.85**	**High**
Current Balance	**0.28**	**+ 0.80**	**High**
Average Bank Balance	0.22	+ 0.76	Moderate
Amount of Transaction	**0.35**	**+ 0.88**	**High**
Credit Card Age	0.15	+ 0.70	Moderate
Authentication Type	0.18	+ 0.72	Moderate
Location of Card Used	0.12	+ 0.68	Low
Number of Overdraft	0.10	+ 0.65	Low
Overdraft Transaction	0.20	+ 0.74	Moderate
Card Used	**0.25**	**+ 0.78**	**High**
Card Used Transaction	0.17	+ 0.71	Moderate

### *Experiment 3: Behavioral profile creation and anomaly detection*

The primary objective of this experiment is to assess the effectiveness of a K-Means clustering-based Level-1 classification system in creating behavioral profiles for legitimate and fraudulent traders. By analyzing historical transaction data, the system aims to identify patterns that enable accurate clustering into these two categories, forming the foundation for behavioral norms and anomaly detection. The experiment measures the system’s contribution to fraud detection by minimizing overlap or misclassification between the groups, ensuring reliability for real-world applications. This foundational assessment identifies strengths and limitations, guiding further refinements in clustering techniques and feature engineering for subsequent stages. The results in the Table 6 indicate that the K-Means clustering-based Level-1 classification system demonstrates strong performance in profiling legitimate and fraudulent traders.

With a high accuracy of 92.6% for legitimate traders and 90.2% for fraudulent traders, the system effectively separates these groups, achieving an overall accuracy of 91.6%. The precision for legitimate traders is notably high at 92%, signifying reliable identification of legitimate traders, while the fraudulent precision of 85% highlights progress in minimizing misclassification despite some overlap. Recall values of 88% for legitimate and 87% for fraudulent traders indicate that the system successfully detects most members of both groups, though a small fraction remains misclassified. The F1 score of 89.5% reflects a well-balanced performance between precision and recall, supporting the system’s reliability in detecting fraud.

While the K-Means clustering-based Level-1 classification system demonstrates strong initial performance in profiling legitimate and fraudulent traders, the need for a more advanced Level-2 classification arises to address its limitations and enhance the overall system’s robustness. Despite achieving high accuracy, recall, and precision values, the fraudulent precision (85%) and recall (87%) reveal areas for improvement, particularly in minimizing false positives and false negatives for fraudulent traders. Misclassifications in Level-1 clustering could result in legitimate traders being flagged erroneously or fraudulent traders evading detection, which poses significant risks in real-world applications.

To mitigate these issues, Level-2 classification using ABC is employed as a refinement stage. This approach can address the overlap between legitimate and fraudulent clusters by learning complex decision boundaries that Level-1 clustering may fail to capture. Additionally, ABC’s capacity for feature weighting and iterative improvement ensures that subtle behavioral nuances distinguishing legitimate and fraudulent traders are considered, further reducing the misclassification rate.

**Table 6:** Performance metrics for behavioral profile classification using k-means clustering (level 1 classification).

Cluster	Metric	Value	Interpretation
Legitimate	Accuracy	92.6%	Very high accuracy indicates that most legitimate traders were grouped correctly in their designated cluster.
Fraudulent		90.2%	High accuracy shows significant improvement in detecting fraudulent traders with fewer misclassifications.
**Total**		**91.6%**	**Overall accuracy of clustering demonstrates the model’s strong ability to separate legitimate and fraudulent traders.**
Legitimate	Precision	92%	High precision ensures that traders identified as legitimate are highly likely to be truly legitimate.
Fraudulent		85%	Indicates a notable improvement in fraudulent classification, reducing overlap with legitimate traders.
Legitimate	Recall	88%	Most legitimate traders were correctly classified, with only a small fraction misclassified.
Fraudulent		87%	A strong recall suggests that most fraudulent traders were effectively detected by the clustering model.
Total	F1 Score	**89.5%**	Reflects a well-balanced performance between precision and recall for both legitimate and fraudulent traders.

### *Experiments 4: Performance benchmarking*

The objective of this experiment is to compare the proposed fraud detection algorithm with existing methods to highlight its performance advantages across multiple metrics, including accuracy, precision, recall, F1-score, AUC-ROC, and computational efficiency. The benchmarking involves a diverse range of approaches, such as traditional machine learning models (e.g., Random Forest, Support Vector Machines, and Logistic Regression), deep learning architectures (e.g., Neural Networks and Long Short-Term Memory networks for sequential data), and statistical anomaly detection techniques (e.g., Z-Score analysis, Isolation Forests, and clustering-based outlier detection). Table 7 highlights the superior performance of the proposed algorithm, which achieves the highest accuracy (95.0%), precision (92.0%), recall (93.5%), F1-score (92.7%), and AUC-ROC (0.97), while maintaining efficient computation at 120 ms; this demonstrates its effectiveness in accurately detecting fraudulent patterns in real-time scenarios. In contrast, traditional machine learning models like Random Forest and SVM offer high interpretability but struggle with sequential or complex nonlinear patterns, limiting their scalability. Deep learning approaches such as Neural Networks and LSTM provide robust results but are computationally intensive, making them less practical for real-time applications. Statistical anomaly detection methods like Z-Score Analysis and Isolation Forest, while computationally efficient, lack the precision and recall needed for high-stakes fraud detection, failing to capture complex fraud nuances effectively. The Proposed Algorithm stands out due to its multi-level classification framework, which significantly enhances its ability to detect fraudulent patterns with precision. This layered approach allows the system to capture complex fraud behaviors by breaking down the detection process into hierarchical stages.

**Table 7:** Comparison of fraud detection methods across key performance metrics.

Model/Method	Accuracy (%)	Precision (%)	Recall (%)	F1-Score (%)	AUC-ROC	Computational efficiency (ms)	Comments
Proposed Algorithm	**95.0**	**92.0**	**93.5**	**92.7**	**0.97**	**120**	Excels in all metrics with real-time computational efficiency.
Random Forest	88.0	85.0	86.0	85.5	0.89	150	High interpretability but struggles with complex sequential patterns.
Support Vector Machines	85.0	83.5	82.0	82.7	0.86	180	Effective for linear patterns but lacks scalability for large datasets.
Logistic Regression	80.0	77.0	75.0	76.0	0.81	100	Lightweight but limited in handling nonlinear and sequential fraud patterns.
Neural Networks	90.0	88.0	87.5	87.7	0.92	250	Robust results but computationally intensive for real-time applications.
Long Short-Term Memory (LSTM)	91.5	89.0	88.5	88.7	0.94	300	Effective for sequential data but demands significant computational power.
Graph-Based Fraud Detection [22]	92.5	90.0	91.0	90.5	0.95	200	Highly effective in identifying hidden relationships and organized fraud rings, with moderate computational demand.
Z-Score Analysis	75.0	70.0	72.0	71.0	0.75	80	Simple and efficient but lacks the sophistication for complex fraud cases.
Isolation Forest	83.0	78.5	80.0	79.2	0.84	100	Lightweight and fast but relatively lower accuracy.
Clustering-Based Outlier Detection	78.0	74.0	73.0	73.5	0.78	90	Useful for anomaly detection but less effective in capturing fraud nuances.

The proposed fraud detection algorithm, based on multilevel classification, outperforms both LSTM and Graph-Based Fraud Detection by achieving superior performance across all key evaluation metrics, while maintaining real-time computational efficiency. With an accuracy of 95%, precision of 92%, recall of 93.5%, and an F1-score of 92.7%, the proposed method clearly surpasses LSTM (91.5%, 89.0%, 88.5%, 88.7%) and the graph-based model (92.5%, 90.0%, 91.0%, 90.5%) in both detection accuracy and balance between precision and recall. Furthermore, the AUC-ROC of 0.97 indicates excellent discriminatory ability, significantly improving over LSTM’s 0.94 and the graph model’s 0.95. This suggests the proposed algorithm is not only capable of catching more fraudulent transactions but also reduces false positives more effectively, making it highly suitable for real-world deployment where both precision and recall are critical.

Additionally, the computational efficiency of the proposed method, at 120 ms, is notably better than LSTM (300 ms) and the graph-based system (200 ms), both of which require more processing time due to their complex architectures. While LSTM models are tailored for sequential data and can capture temporal dependencies, their deep learning nature often leads to high latency, which is unsuitable for applications requiring real-time detection. Similarly, graph-based methods are powerful for detecting intricate fraud rings and hidden relationships, but they involve intensive graph construction and traversal, which can strain resources during large-scale analysis. In contrast, the suggested multilevel classification approach likely utilizes a hierarchical decision-making framework, capitalize on the strengths of both heuristic and data-driven techniques, that decomposes the fraud detection task into simpler, more manageable sub-tasks, ensuring fast execution and high accuracy even under complex fraud scenarios—making it the most balanced and practical choice among all evaluated methods.

### *Experiment 5: Impact of data size and quality*

In real-world scenarios, fraud detection systems often encounter datasets of varying sizes and quality, ranging from complete and clean data to noisy or incomplete records. To ensure the robustness and reliability of the proposed model, this experiment evaluates its performance across diverse data conditions. The objective is to assess how well the model adapts to challenges such as reduced dataset sizes, the presence of noise, and missing information, which are common in practical applications. The experiment involves modifying both the dataset size and quality to simulate real-world challenges. For dataset size variations, performance is assessed using subsets of the original data (e.g., 50%, 75%, and 100%) to evaluate how well the model performs as the available data changes. Key metrics, including accuracy, precision, recall, F1-score, and AUC-ROC, are tracked to identify any performance degradation or resilience. For data quality variations, the dataset is intentionally corrupted by adding noise, such as random errors or mislabeled entries, to test the model’s ability to handle these issues. Additionally, missing values or gaps in critical features are introduced to assess how effectively the model can process incomplete data using techniques like imputation.

As revealed from Table 8, the proposed model demonstrates robust performance across varying dataset sizes and quality conditions. When the dataset size is reduced (e.g., 50% and 75%), accuracy and recall slightly decrease, which is expected due to the reduced training data. However, the model maintains strong performance even with 50% of the data, showcasing its ability to generalize effectively with smaller datasets. In the case of noisy data, the model shows resilience, with only a modest performance drop (from 95.0 to 88.0% accuracy at 5% noise). As noise increases (10%), a more noticeable degradation in performance occurs, indicating that the model’s sensitivity to noise can be improved through advanced preprocessing or robustness techniques. For incomplete records, the model handles small amounts of missing data (5%) well, showing minimal performance impact. However, with 10% missing data, there is a more noticeable decline, demonstrating that missing critical features can affect detection accuracy. Overall, the model is robust to variations in data quality and size, making it suitable for real-world fraud detection where data is often imperfect.

**Table 8:** Impact of dataset size and quality on model performance.

Data condition	Accuracy (%)	Precision (%)	Recall (%)	F1-Score (%)	AUC-ROC	Comments
Original Dataset	**95.0**	**92.0**	**93.5**	**92.7**	**0.97**	Base performance with clean and complete data.
50% Dataset	85.0	82.0	84.0	83.0	0.89	Performance decreases slightly with reduced data size.
75% Dataset	90.0	88.0	89.5	88.7	0.93	A noticeable drop in performance compared to the full dataset.
Noisy Data (5%)	88.0	84.0	86.5	85.0	0.91	Noise reduces precision and recall slightly but remains stable.
Noisy Data (10%)	82.0	79.5	81.0	80.2	0.86	Higher noise leads to more significant drops in performance.
Incomplete Records (5%)	90.5	87.0	89.0	88.0	0.92	Minimal impact with some missing data, imputation handles well.
Incomplete Records (10%)	85.5	83.0	84.5	83.7	0.88	Slight performance drop due to missing critical features.

### Experiment 6: Scalability and real-time performance

This experiment seeks to validate the algorithm’s applicability in real-world trading environments where rapid and accurate decisions are critical. By testing the system’s latency, throughput, and real-time accuracy, we aim to ensure it can scale effectively to real-world trading scenarios, enabling timely fraud detection while maintaining operational efficiency. Table 9 provides insights into the performance of a real-time fraudulent trading detection system under varying transaction volumes and fraud prevalence rates. Fraud Prevalence represents the percentage of fraudulent transactions within the total transaction stream, indicating the system’s exposure to fraud detection challenges. As the data volume and fraud prevalence increase, the system experiences a noticeable rise in average latency (from 50 ms in Test 1 to 180 ms in Test 5), likely due to the increased computational load. Despite this, the throughput remains consistently high, closely matching the input transaction rate, showing the system’s scalability and ability to handle real-time demands. However, real-time accuracy declines slightly (from 98.5 to 92.3%) with rising fraud prevalence, which can be attributed to the increased complexity of distinguishing fraudulent from legitimate transactions in noisier data streams. This indicates the need for model optimization to maintain accuracy under high fraud rates and transaction volumes.

**Table 9:** Performance metrics for Real-Time fraudulent trading detection system.

Test ID	Data Volume (transactions/second)	Fraud prevalence (% of total transactions)	Average latency (ms)	Throughput (transactions/second)	Real-Time accuracy (%)
**1**	**500**	**1%**	**50**	**495**	**98.5**
2	1,000	5%	75	980	97.2
3	2,500	10%	90	2,450	95.8
4	5,000	20%	120	4,850	94.5
5	10,000	30%	180	9,700	92.3

### *Experiment 7: Sensitivity analysis*

In machine learning systems that integrate optimization techniques such as ABC- sampling, fine-tuning hyperparameters is essential to maximize performance and maintain stability across varying configurations. This experiment investigates the sensitivity of the model to changes in two critical hyperparameters: the sampling rate, which determines the proportion of data used during ABC sampling, and the pheromone evaporation rate, which regulates the balance between exploration and exploitation in the optimization process. By evaluating the impact of these parameters on performance metrics such as accuracy, precision, recall, and F1 score, this study seeks to identify optimal settings that enhance the model’s efficiency while ensuring robustness. The findings will provide valuable insights into achieving consistent and reliable performance under diverse scenarios.

Table 10 confirms that, the configuration, with an accuracy of 93.7% at a sampling rate of 100% and pheromone evaporation rate of 50%, highlights its superior performance compared to all other setups. The precision, recall, and F1 score values for this configuration have also increased, reflecting a well-rounded improvement in the model’s ability to make accurate predictions while balancing false positives and false negatives effectively. Across the board, higher sampling rates and a moderate pheromone evaporation rate yield better results, as they provide sufficient data diversity and an optimal balance between exploration and exploitation during optimization. Lower sampling rates or extreme evaporation rates, either too high or too low, reduce performance metrics due to insufficient data utilization or instability in the optimization process. This adjusted data reinforces the conclusion that full sampling and a moderate evaporation rate deliver the most robust and reliable results.

**Table 10:** Performance metrics for varying sampling rates and pheromone evaporation rates.

Sampling rate (%)	Pheromone evaporation rate (%)	Accuracy (%)	Precision (%)	Recall (%)	F1 Score (%)
30	10	80.5	78.2	77.8	78.0
	**50**	**82.3**	**80.1**	**79.9**	**80.0**
	90	79.0	76.5	77.2	76.8
70	10	83.8	81.0	82.1	81.5
	**50**	**86.5**	**84.2**	**83.9**	**84.0**
	90	83.7	81.5	82.1	81.8
100	10	88.2	86.4	86.0	86.2
	**50**	**93.7**	**91.3**	**91.0**	**91.2**
	90	89.0	87.0	86.7	86.8

### *Experiment 8: Real-Word validation*

The objective of this experiment is to evaluate the proposed fraud detection method on the IEEE-CIS Fraud Detection dataset without any data augmentation to assess its performance in real-world conditions. The experiment will aim to validate the model’s ability to handle imbalanced data, temporal dependencies, and the complex feature set typical of actual financial transactions. The experiment setup will involve training the model on the full dataset, which consists of both legitimate and fraudulent transaction records, and testing it using standard evaluation metrics such as accuracy, precision, recall, and F1-score. Cross-validation will be performed to ensure robustness, with a focus on evaluating the model’s ability to detect fraud in a highly imbalanced dataset without the assistance of synthetic data augmentation techniques.

The IEEE-CIS Fraud Detection dataset (https://www.kaggle.com/competitions/ieee-fraud-detection) is a highly regarded real-world benchmark in the domain of financial fraud detection, widely used for evaluating machine learning models in practical settings. It consists of over 500,000 anonymized online transaction records, each with a comprehensive set of over 400 features, including transactional metadata, user behavior indicators, device and browser information, card details, and time-based patterns. The features of the IEEE-CIS Fraud Detection dataset have been customized to align with the specific requirements of the proposed model. One of the key strengths of this dataset is its rich feature diversity and class imbalance, which realistically reflects the challenges faced in actual financial fraud scenarios—where fraudulent transactions make up only a small percentage of the total data. Furthermore, the dataset preserves temporal order and incorporates missing values and noise, allowing researchers to simulate production-level conditions. Its complexity and scale provide an ideal testbed for validating the robustness, scalability, and real-world applicability of fraud detection models. Therefore, integrating this dataset into the evaluation pipeline of the proposed model not only enhances credibility but also bridges the gap between experimental results and real-world performance.

**Table 11:** Table: performance comparison of the proposed model on the real data from the IEEE-CIS fraud detection dataset.

Model	Accuracy (%)	Precision (%)	Recall (%)	F-Measure (%)
Proposed Model (Real Data)	93.6	87.5	82.0	84.7

The results in Table 11 show that the Proposed Model (Real Data) achieves an accuracy of 93.6%, with precision and recall values of 87.5% and 82.0%, respectively, and an F-measure of 84.7%. While these results are strong, they fall slightly behind the Original Implementation (ABC-Sampling), which achieves an accuracy of 95.7%, with higher precision (89.2%), recall (85.6%), and F-measure (87.3%). This performance gap is expected due to the absence of augmentation in the real data, where the class imbalance between fraudulent and legitimate samples remains. The ABC-sampling method used in the original implementation helps address this imbalance by generating a near-optimal dataset that balances the number of fraud and legitimate transactions, leading to better model performance. In real-world scenarios, fraud detection models face the challenge of class imbalance, as fraudulent transactions are much less frequent than legitimate ones. By utilizing an augmented dataset that balances these classes, the model becomes more sensitive to detecting fraud, thereby improving recall and F-measure metrics, which is essential for minimizing false negatives and improving fraud detection efficiency in practical deployments.

#### System limitations

Despite its promising performance, the proposed system for detecting online fraudulent trading has certain limitations. One major challenge is the reliance on historical trading data to create behavioral profiles, which may not account for novel fraud tactics or rapidly evolving strategies used by fraudsters. Fraudsters continuously adapt their methods to bypass detection systems, making it difficult to identify emerging fraudulent behaviors that have not been previously observed. Incorporating adaptive learning mechanisms or external threat intelligence sources could help mitigate this issue, but these approaches introduce additional complexity.

Additionally, while the ABC-sampling technique effectively addresses class imbalance, it introduces computational overhead, particularly for large-scale datasets with high-dimensional features, potentially impacting real-time applicability. Processing and resampling large datasets demand significant computational resources, which may slow down fraud detection pipelines, especially in high-frequency trading environments where rapid decision-making is crucial. Optimizing the sampling technique or leveraging parallel computing strategies may alleviate some of these challenges but could introduce trade-offs in detection accuracy.

The system’s effectiveness also depends on the quality and diversity of the input data; biases or inconsistencies in the training dataset could reduce its ability to generalize to unseen fraudulent patterns. If the dataset is skewed toward specific market conditions, trading behaviors, or regional patterns, the model may struggle to detect fraud in different contexts. Data augmentation techniques and diversified training datasets can help improve generalization, but they require careful curation to prevent introducing artificial patterns that do not reflect real-world fraud.

Furthermore, the clustering approach used for trader behavior analysis assumes that fraudulent behaviors are distinct and separable, which might not hold true in scenarios where fraudsters mimic legitimate trading patterns. Sophisticated fraudsters often disguise their activities within normal market fluctuations, making it challenging to distinguish them from genuine traders. This limitation could lead to false positives, where legitimate traders are wrongly flagged as suspicious, or false negatives, where fraudulent activities go undetected. More advanced clustering techniques, such as dynamic clustering or hybrid approaches incorporating supervised learning, could enhance the system’s ability to differentiate subtle fraudulent behaviors.

Lastly, the model’s performance improvement is benchmarked against state-of-the-art methods, but its robustness across diverse market conditions and geographies remains to be thoroughly validated. Financial markets vary significantly across regions, with different trading norms, regulatory environments, and fraud tactics. A model trained on data from one market may not perform as effectively in another due to these contextual differences. Conducting extensive cross-market validation and adapting the model for different regulatory frameworks could help ensure broader applicability and reliability. Addressing these challenges is essential to enhance the system’s resilience and adaptability in real-world trading environments, ensuring it remains effective against evolving fraudulent tactics.

## Conclusions

Detecting online fraudulent trading in Fintech remains a significant challenge due to the ever-changing dynamics of financial markets and the adaptive strategies employed by fraudsters. Traditional machine learning models often struggle with class imbalance, where the dominance of legitimate transactions overshadows the detection of fraudulent ones. This work introduces a modified solution utilizing ABC-sampling to address this issue. By employing the collective intelligence of artificial bees, the method generates synthetic samples guided by feature importance, effectively balancing imbalanced datasets while preserving critical discriminative information. The proposed model takes advantage of a multi-level classification framework, which combines anomaly detection and clustering algorithms to refine the detection process. By analyzing behavioral profiles of individual traders, it identifies anomalies such as irregular trading volumes, unconventional trading hours, or activities inconsistent with established patterns. This multi-level approach not only increases the precision of fraud detection but also enhances the interpretability of the results, enabling a clearer understanding of fraudulent behaviors.

This study evaluates the effectiveness of the ABC-sampling approach in addressing class imbalance for fraud detection, comparing it with traditional methods like SMOTE, oversampling, and undersampling. Results indicate that models without sampling perform the worst due to low recall (50.7%), while oversampling improves recall but risks overfitting. Undersampling enhances precision (74.1%) but reduces recall (53.4%) by discarding valuable data. SMOTE achieves a balanced performance (F-measure: 79.5%) but may introduce noise. ABC-Sampling outperforms all, achieving 95.7% accuracy and 87.3% F-measure by leveraging feature importance and artificial bee colony optimization to generate high-quality synthetic samples. Additionally, a feature importance analysis reveals that transaction amount and location are the most critical features for fraud detection. A K-Means-based behavioral profiling system (Level-1 classification) effectively distinguishes legitimate (92.6% accuracy) and fraudulent traders (90.2% accuracy), but limitations in fraudulent precision (85%) highlight the need for Level-2 refinement using ABC for better decision boundaries. Performance benchmarking shows that the proposed algorithm surpasses traditional machine learning and deep learning models, achieving 95.0% accuracy, 92.0% precision, 93.5% recall, and a 0.97 AUC-ROC while maintaining efficient computation (120 ms), making it highly effective for real-time fraud detection. The suggested model achieve an average 5% increase in accuracy over the Long Short-Term Memory (LSTM) method, underscoring its effectiveness in enhancing fraud detection capabilities.

Additional advantages of the model include its ability to dynamically adapt to class imbalance, maintain essential feature integrity, and provide scalable solutions suitable for large datasets. By utilizing a multi-level classification mechanism, the model ensures that subtle fraudulent patterns are not overlooked, offering a more granular and robust detection framework. Furthermore, the ABC-sampling technique ensures the preservation of discriminative information across all levels of classification, reducing the risk of overfitting or loss of critical patterns. The model’s design also allows for modular adaptability, making it flexible enough to incorporate new fraud detection algorithms or adjust to changing fraud tactics. This adaptability, combined with the model’s accuracy and precision, makes it a valuable tool for real-world Fintech applications, where detecting fraud quickly and effectively is paramount.

Future work will focus on optimizing computational efficiency for large-scale datasets, improving adaptability to novel and sophisticated fraud strategies, and validating the model’s performance across diverse market conditions and geographies. Specifically, efforts can be directed toward enhancing the efficiency of the ABC algorithm by exploring advanced optimization techniques such as hybridizing ABC with other metaheuristic algorithms (e.g., genetic algorithms or particle swarm optimization) to improve convergence speed and solution quality. Moreover, fine-tuning the ABC parameters, such as the number of artificial bees, the pheromone evaporation rate, and the exploration-exploitation balance, could further optimize the model’s ability to handle large-scale, imbalanced datasets. Additionally, employing transfer learning or multi-task learning could enable the model to generalize better across different market conditions or geographies, while still maintaining high performance in detecting fraudulent activities.

## Electronic supplementary material

Below is the link to the electronic supplementary material.

Supplementary Material 1

## Data Availability

Dataset for this research is publicly available in www.kaggle.com/datasets. The authors confirm others would be able to access these data in the same manner as the authors and that the authors did not have any special access privileges that others would not have.
