# Supplementary material for: Intelligent approach to detecting online fraudulent trading with solution for imbalanced data in fintech forensics
Source: Sci Rep. 2025 May 23;15:17983. doi: 10.1038/s41598-025-01223-8 (PMC12102310; doi:10.1038/s41598-025-01223-8)
Supplement: Supplementary file 1 — Supplementary Material 1 [file 41598_2025_1223_MOESM1_ESM.docx]

Appendix

| Algorithm 1: **ABC Sampling Technique for Balancing Imbalanced Datasets** |
| --- |
| **Input:**  Dataset $D=\{(X_{i},y_{i}) \mid X_{i}\in\mathbb{R}^{d},y_{i}\in\{0,1\}\}$  $D_{1}$ ​: Minority class (fraudulent transactions)  $D_{0}$: Majority class (legitimate transactions)  Target ratio $R_{target}$  Parameters: $N$(number of food sources), *limit* ($max\_ trials$), *ϕ* (exploration factor)  **Output:**  Balanced dataset $\hat{D}$  **Algorithm Steps**   1. **Initialization**:  - Extract $D_{1}$​ from $D$ (minority class). - Initialize $N$ food sources $X=\{X_{1},X_{2},\ldots,X_{N}\}$, where each $X_{i}$ is randomly selected from $D_{1}$​. - Compute the centroid of $D_{1}$:   ${Centroid}_{fraud}=\frac{1}{\left\vert D_{1} \right\vert}\sum_{X_{i}\in D_{1}} X_{i}$   1. **Define Fitness Function:**  - For each food source $X_{i}$   $F\left( X_{i} \right)=\frac{1}{1+dist\left( X_{i},{Centroid}_{fraud} \right)}$  (e.g., using Euclidean distance).   1. **Repeat Until** $\left\vert D_{1} \right\vert/\left\vert D_{0} \right\vert\boldsymbol{\geq}R_{target}$  - **Employed Bee Phase**:   **For each** food source $X_{i}$:   1. Generate a new candidate $V_{ij}$   $V_{ij}=X_{ij}+\phi_{ij}\left( X_{ij}-X_{kj} \right)$  $X_{k}$ is randomly selected from $D_{1}$​, $k\neq i$, and$\phi_{ij}\in[-1,1]$ is a random number.   1. Evaluate $F(V_{ij})$:  - **If** $F\left( V_{ij} \right)>F\left( X_{i} \right),\mathrm{update}X_{i}=V_{ij}$. - **Onlooker Bee Phase:**  1. Calculate selection probability for each food source $X_{i}$   $P_{i}=\frac{F\left( X_{i} \right)}{\sum_{j=1}^{N} F\left( X_{i} \right)}$   1. Select food sources based on$P_{i}$​. 2. Repeat the steps of the employed bee phase for selected sources.  - **Scout Bee Phase:**   **For each** $X_{i}$:   1. If $X_{i}$does not improve after *limit* trials:  - Abandon $X_{i}$​. - Replace $X_{i}$​ with a new transaction randomly initialized in the feature space.  1. **Stopping Criteria:**  - Stop the process when the size of the minority class $\left\vert D_{1} \right\vert$ satisfies:   $\left\vert D_{1} \right\vert/\left\vert D_{0} \right\vert\boldsymbol{\geq}R_{target}$   1. **Combine Data:**   Combine the synthetic transactions with the original dataset to form $\hat{D}$.  **Return:** $\hat{D}$, the balanced dataset. |

| **Algorithm 2: Rule-based filtering (initial classifier)** |
| --- |
| **Input**: Transaction’s attributes for each customer $A_{T_{i,j}}$  The five filtered parameters for each customer’s transaction ${FP}_{T_{i,j}}$  $A_{C_{i}}$// *computed according to Table 3*  **Output**: The initial transaction’s classification $\xi_{T_{i,j},L_{0}}$  *// level 0 classification*  **Begin**  - **IF** ${CC}_{Frquency}<0.2$ **THEN** not fraud, $\delta_{1}={CC}_{Frquency}$  *// The threshold of* $0.2$ *represents a low frequency of card usage, which is considered typical or non-suspicious*  *behavior for certain cardholders*  - **IF** $N_{T\_cu}>\left( 5\times{CC}_{Frquency} \right)$ **THEN** fraud, $\delta_{1}=N_{T\_cu} \times{CC}_{Frquency}$  *// The rule sets a threshold that triggers fraud detection when the current transaction count* $N_{T\_cu}$ *exceeds five*  *times the typical frequency*  - **IF** ${CC}_{loc}\leq5$ **THEN** not fraud, $\delta_{2}=0.01$  *// The threshold of 5 indicates that using a card in five or fewer distinct locations is considered normal or typical behavior for the cardholder*  - **IF** $T_{loc}>5\times{CC}_{loc}$ **THEN** fraud, $\delta_{2}={CC}_{Usage\_Location}$  // *The threshold is set at five times the cardholder's normal location diversity. If the current number of locations exceeds this value, the activity is flagged as suspicious.*  - **IF** $N_{cc\_od}\leq0.2$ **THEN** not fraud, $\delta_{3}={CC}_{Overdraft}$  *// The threshold*$0.2$ *indicates a very low frequency of credit limit exceedance. Values at or below this level suggest that the cardholder has rarely, if ever, gone over their credit limit.*  - **IF** $T_{od}=true$ $Fraud condition number of locations Card used Today$**THEN** fraud, $\delta_{3}=T_{od} {\times CC}_{Overdraft}$  *// suggests the cardholder is spending funds they don’t have access to, which could signal fraudulent activity, especially if it deviates from their usual behavior*  - **IF** ${CC}_{cb}\leq0.25$ **THEN** not fraud, $\delta_{4}={CC}_{cb}$  *// A remaining balance at or below 25% suggests the cardholder has already used a significant portion of their credit limit*  - **IF** $T_{od}=true$ **THEN** fraud, $\delta_{4}={CC}_{cb}\times{CC}_{Balance}$  *// Check whether overdraft condition occurred today*  - **IF** ${(10\times CC}_{Daily\_Spending})>T_{amt}$ $Fraud condition number of locations Card used Today$**THEN** fraud,  $\delta_{5}=T_{amt}/\left( {10\times CC}_{Daily\_Spending} \right)$  **ELSE** not fraud, $\delta_{5}=0.01$  *// If a transaction’s amount exceeds ten times the cardholder’s average daily spending, it is considered an anomaly and flagged as potentially fraudulent*  Return $\xi_{T_{i,j},L_{0}}$, ${\delta_{T_{i,j}}=\left\{ \delta_{1,T_{i,j}}, \delta_{2,T_{i,j}}, \delta_{3,T_{i,j}}, \delta_{4,T_{i,j}}, \delta_{5,T_{i,j}} \right\}}$  *//* $T_{i,j}$ *transaction j for customer i* |

| **Algorithm 3:** ABC-Based Level 2 Classification for K-Means |
| --- |
| 1. **Initialization**:  - Input the dataset $D$ with each transaction represented as a vector of 7 Boolean values: $\left[ \delta_{1,T_{i,j}}, \delta_{2,T_{i,j}}, \delta_{3,T_{i,j}}, \delta_{4,T_{i,j}}, \delta_{5,T_{i,j}}, {}_{Model,T_{i,j}, l1}, {}_{Model,real} \right]$ - Define the number of clusters $K=2$ and the number of artificial bees (employed, onlooker, scout). - Randomly initialize $K$centroids$C_{k}$ within the Boolean feature space.  1. **Fitness Function:**   Define the fitness function based on:   - Intra-cluster similarity: Minimize the distance between data points and their assigned centroids. - Classification alignment: Maximize the match between ${}_{Model,T_{i,j}, l1}$ and ${}_{Model,real}$ for each cluster.   $Fitness=\alpha\times Intra-Cluster Similarity+\beta\times Classification Alignment$  $\alpha$ and$\beta$ are weighting factors balancing similarity and alignment.   1. **Employ Bees:**  - Each employed bee explores the neighborhood of its current solution (centroid) to find a better centroid position. - Update centroids $C_{k}$​ based on the exploration.  1. **Calculate Probabilities:**  - Calculate the probability $P_{k}$​ of selecting a cluster centroid based on its fitness:   $P_{k}=\frac{Fitness}{\sum_{i=1}^{k} {Fitness}_{i}}$   1. **Onlooker Bees:**  - Assign onlooker bees to centroids based on $P_{k}$. - Each onlooker bee refines centroids by exploiting promising solutions (clusters with high fitness).  1. **Scout Bees:**  - Identify stagnating centroids (not improving over iterations) and replace them with new random centroids in the feature space.  1. **Cluster Assignment:**  - Assign each data point to the nearest centroid using a similarity measure (e.g., Hamming distance for Boolean features). - Evaluate the clustering result using the fitness function.  1. **Termination:**  - Repeat Steps 3–7 for a predefined number of iterations or until the centroids converge. - Output the optimized centroids $C_{k}$ and the final clusters.  1. **Level 2 Classification:**  - Based on the final clusters, assign a classification label ${}_{Model,T_{i,j}, l1}$ for new data points by majority voting of ${}_{Model,real}$ values within each cluster. |
